# Co-infection with Zika and Dengue Viruses in 2 Patients, New Caledonia, 2014

**DOI:** 10.3201/eid2102.141553

**Published:** 2015-02

**Authors:** Myrielle Dupont-Rouzeyrol, Olivia O’Connor, Elodie Calvez, Maguy Daurès, Michéle John, Jean-Paul Grangeon, Ann-Claire Gourinat

**Affiliations:** Institut Pasteur, Noumea, New Caledonia (M. Dupont-Rouzeyrol, O. O’Connor, E. Calvez, A.-C. Gourinat);; Direction des Affaires Sanitaires et Sociales, Noumea (M. Daurès, M. John, J.-P. Grangeon)

**Keywords:** Zika virus, dengue virus, viruses, co-infection, emerging diseases, detection, New Caledonia

**To the Editor:** Dengue is the most prevalent arthropod-borne viral disease in tropical and subtropical countries. Every year, dengue virus (DENV) infections cause more than 50 million cases, 500,000 hospitalizations, and 12,500 deaths worldwide (*1*). DENV belongs to the genus *Flavivirus* and is transmitted by *Aedes* spp. mosquitoes. There are 4 distinct serotypes (DENV-1 to DENV-4), and infection with 1 serotype does not provide long-term, cross-protective immunity against the other 3 serotypes.

In New Caledonia, DENV outbreaks have occurred since World War II and have been caused mainly by 1 serotype/genotype introduced from a country to which dengue is hyperendemic. Since 2000, New Caledonia has had recurrent DENV-1 outbreaks (*2*). In 2014, circulation of DENV-1 and DENV-3 was still reported in this country (*3*).

Zika virus (ZIKV) is an emerging mosquito-borne virus that belongs to the genus *Flavivirus* and was first isolated in Uganda (*4*). ZIKV is believed to be transmitted to humans by *Aedes* spp. mosquitoes. Before 2007, few human cases of infection had been reported. In 2007, the first Zika epidemic occurred in Yap, Federated States of Micronesia (*5*). In October 2013, a ZIKV outbreak was reported in French Polynesia (*6*).

In New Caledonia, the first cases of ZIKV infection imported from French Polynesia were confirmed at the end of November 2013, and the first autochthonous cases were reported by mid-January 2014. Early in February 2014, the New Caledonia Health Authority declared an outbreak situation. Since February 2014, a total of 1,385 ZIKV laboratory-confirmed cases have been detected, including 35 imported cases (32 from French Polynesia, 2 from Vanuatu, and 1 from the Cook Islands). Concomitant with this ZIKV outbreak, circulation of DENV-1 and DENV-3 was reported; >150 cases were biologically confirmed during January–September 2014 (*3*). Thus, New Caledonia currently has 3 arboviruses co-circulating.

In recent years, co-circulation of multiple DENV serotypes or DENV and chikungunya virus has been reported. Although rare co-infections have been identified (*7**,**8*), given the similar clinical features and lack of concurrent testing, co-infections might not be identified. We report detection of ZIKV and DENV genomes in serum of a traveler (patient 1) who returned from French Polynesia where ZIKV and DENV were co-circulating (*6**,**9*) and in serum of a person (patient 2) in New Caledonia who had no travel history. The traveler was co-infected with ZIKV and DENV-3, and the local patient was co-infected with ZIKV and DENV-1.

Patient 1 was a 14-year-old boy who had fever (39.5°C), headache, arthralgia, asthenia, and myalgia. No hemorrhagic or neurologic findings were reported, and the patient recovered within 3 days. A complete blood count showed mild thrombocytopenia (platelet count 129 × 10^9^ platelets/L; reference range 150–400 × 10^9^ platelets/L), leukopenia (leukocyte count 2.75 × 10^9^ cells/L; reference range 4–10 × 10^9^ cells/L) with associated stimulated lymphocytes, and discreet hepatic cytolysis (aspartate aminotransferase 55 IU/L; reference value <34 UI/L, and alanine aminotransferase 51 IU/L; reference value <55 IU/L). Serum was analyzed by using real-time reverse transcription PCR (RT-PCR) and was positive for ZIKV as recommended by Lanciotti et al. (*5*) and DENV-3.

Patient 2 was a 38-year-old woman who had fever (40°C), headache, arthralgia, asthenia, myalgia, retroorbital pain, conjunctivitis, diarrhea, nausea, and a diffuse pruritic maculopapular rash. No hemorrhagic or neurologic findings were reported, but signs of illness lasted ≈1 week. The patient had mild thrombocytopenia (platelet count 123 × 10^9^ platelets/L) and leukopenia (leukocyte count 2.65 × 10^9^ cells/L). Serum was analyzed by using RT-PCR (*5*) and was positive for ZIKV and DENV-1.

Co-infections were assessed by sequencing partial ZIKV membrane–envelope gene regions for isolates (GenBank accession nos. KM212963 and KM212967) from both patients, partial DENV-1 envelope gene for an isolate (KM212960) from patient 2, and partial DENV-3 nonstructural protein 5 gene for an isolate (KM212962) from patient 1. Sequencing was conducted at La Plateforme du Vivant (Noumea, New Caledonia).

DENV-1 sequence obtained belonged to genotype I and clustered with DENV-1 sequences isolated in New Caledonia (*2*). DENV-3 sequence obtained belonged to genotype III, similar to DENV-3 recently isolated in French Polynesia (*9*). ZIKV sequences obtained belonged to the Asian lineage and had 99% identity with sequences of ZIKV isolated in French Polynesia in 2013 (*10*).

Serum specimens from both patients were cultured on Vero cells, and supernatants were evaluated by RT-PCR. Each specimen was positive only for DENV, which was probably caused by low viral loads for ZIKV.

We report co-infection of 2 patients with DENV and ZIKV; each patient was infected with a different DENV serotype. No synergistic effects of the 2 viral infections were observed because both patients were not hospitalized and recovered after a mild clinical course.

During this outbreak, patients in New Caledonia were tested for DENV, chikungunya virus, and ZIKV within the framework of the arboviruses sentinel network, which enabled detection of co-infections. Thus, clinicians should be aware of infections with multiple pathogens in the differential diagnosis of dengue-like illness, especially in patients who returned from tropical regions. This diagnostic procedure could be improved by using multiplex RT-PCR for travelers, given the frequent co-circulation of multiple arboviruses in tropical regions.
